# Clinical and laboratory biomarker correlates of lip, oral cavity, and pharyngeal cancer status in a large retrospective clinical database

**DOI:** 10.3389/froh.2026.1888417

**Published:** 2026-08-11

**Authors:** Amr Sayed Ghanem, Róbert Bata, Renáta Jávorné Erdei, Marianna Móré, Attila Csaba Nagy

**Affiliations:** 1 Department of Epidemiology, Institute of Health Sciences, Faculty of Health Sciences, University of Debrecen, Debrecen, Hungary; 2 Department of Health Methodology and Prevention, Institute of Health Sciences, Faculty of Health Sciences, University of Debrecen, Nyíregyháza, Hungary; 3 Institute of Social and Sociological Sciences, Faculty of Health Sciences, University of Debrecen, Nyíregyháza, Hungary

**Keywords:** C-reactive protein, gingival and periodontal disease, head and neck cancer, hemoglobin, inflammatory biomarkers, oral cavity cancer, real-world clinical database, routine laboratory biomarkers

## Abstract

**Introduction:**

Lip, oral cavity and pharyngeal cancer (LOCP) comprises a clinically relevant spectrum of contiguous head-and-neck malignancies, yet large real-world datasets with linked blood data remain uncommon. This study investigated demographic, comorbidity-related, gingival/periodontal, and routine laboratory correlates of recorded ICD-10 C00-C14 LOCP cancer status.

**Methods:**

This patient-level cross-sectional study included 333,807 unique patients from 805,989 clinical records at the University of Debrecen between 2007 and 2022. Descriptive analyses used Pearson's chi-squared and Wilcoxon rank-sum tests. Multivariable logistic regression was applied in four primary models.

**Results:**

Overall, 1,963 patients (0.59%) had recorded LOCP cancer status. In the base clinical model, LOCP cancer status was associated with male sex (OR=3.95, 95% CI: 3.56–4.38), age 45–64 years (OR=10.67, 95% CI: 8.86–12.86), age ≥65 years (OR=7.27, 95% CI: 5.97–8.85), and K05-coded gingival/periodontal disease (OR=8.23, 95% CI: 6.16–10.98). Among biomarkers, log-transformed CRP showed the clearest independent association (OR per 1-SD increase = 1.55, 95% CI: 1.47–1.65), while hemoglobin was inversely associated (OR per 1-SD increase = 0.74, 95% CI: 0.71–0.79). CRP quartiles showed a graded pattern, with Q4 associated with higher odds than Q1 (OR=3.73, 95% CI: 3.01–4.63). Biomarkers modestly improved AUC in complete-case subsets.

**Conclusions:**

Recorded LOCP cancer status was associated with a distinct inflammatory and hematological profile, mainly higher CRP and lower hemoglobin. Longitudinal, site-specific datasets with richer behavioral and tumor-level information are needed before these markers can be interpreted as predictive or etiological indicators.

## 1 Introduction

The World Health Organization International Classification of Diseases, 10th Revision (ICD-10) classifies C00-C14 as malignant neoplasms of the lip, oral cavity, and pharynx, encompassing lip (C00), oral cavity (C01-C06), major salivary glands (C07-C08), tonsil/oropharynx/nasopharynx/pyriform sinus/hypopharynx (C09-C13), and other or ill-defined lip, oral cavity, and pharyngeal sites (C14) (1). This administrative disease block is therefore broader than oral cavity cancer alone and captures a clinically relevant spectrum of contiguous head-and-neck malignancies that remains common globally and in Europe, including Hungary (2–5).

Although these tumors arise locally, their clinical expression is accompanied by measurable systemic disturbance. In head and neck squamous cell carcinoma, elevated pretreatment C-reactive protein (CRP) is associated with poorer outcomes (6, 7), pretreatment anemia or low hemoglobin is a negative survival correlate (8), and elevated red cell distribution width (RDW) has been associated with poorer overall survival in OSCC (9); periodontitis also shows a consistent association with oral squamous cell carcinoma and head and neck squamous cell carcinoma (HNSCC), reinforcing the relevance of chronic inflammatory burden (10, 11).

For this reason, routine laboratory tests deserve attention in real-world databases. CRP, hemoglobin, RDW, and white blood cell count (WBC) are inexpensive and clinically familiar, while glutamate-pyruvate transaminase (GPT), creatinine, and lactate dehydrogenase (LDH) broaden the panel beyond inflammation and erythroid status to wider systemic disturbance (12); importantly, serum-based testing in HNSCC is being actively explored, yet only a limited subset of biomarkers is sufficiently validated for routine use, so these variables are best interpreted as disease-associated correlates that may support case-finding, triage, risk stratification, or monitoring hypotheses after validation rather than as causal or incident-prediction markers (13).

A further rationale for the present study is methodological. Much of the head and neck biomarker literature remains focused on molecular, treatment-specific, or smaller institutional series, whereas large real-world datasets with linked blood data remain comparatively uncommon (13, 14). In Hungary, recent national work has clarified oral-cancer incidence and comorbidity burden, but not integrated routine laboratory phenotyping across the fuller ICD-10 C00-C14 block using the statistical power of a large clinical dataset (15).

We therefore analyzed a large retrospective clinical database to assess demographic, comorbidity-related, gingival/periodontal, and routine laboratory correlates of recorded ICD-10 C00-C14 LOCP cancer status. Specifically, we examined whether inflammatory, hematological, hepatic, and renal biomarkers, including CRP, hemoglobin, RDW, WBC, GPT, creatinine, and LDH, were independently associated with recorded malignant neoplasms of the lip, oral cavity, and pharynx after adjustment for age, sex, entry year, and relevant comorbidities.

## 2 Materials and methods

### 2.1 Study design and data source

This study was designed as a retrospective patient-level cross-sectional analysis using data extracted from the Clinical Center of the University of Debrecen. The source dataset contained 805,989 records from 333,807 unique patients, identified using the anonymized patient identifier. The analytic dataset contained multiple records for some patients within their represented entry year. To avoid non-independence and pseudoreplication, data were aggregated to one row per patient before analysis. Entry year was retained as the calendar year represented for each patient and included as an adjustment variable in regression models to account for calendar-period differences in coding, laboratory testing, and clinical practice. Because exact diagnosis dates and laboratory measurement dates were unavailable, temporal sequencing between laboratory values, comorbidities, and recorded C00-C14 cancer status could not be established; therefore, the analysis was treated as patient-level cross-sectional.

The calendar year of patient entry was recorded using entry year, ranging from 2007 to 2022. Sex was binary with male and female categories. Age was available both as a continuous variable and as a categorical variable, grouped as 0–17, 18–44, 45–64, and ≥65 years. The 18–44-year group was used as the reference category in regression analyses.

### 2.2 Outcome definition

The primary outcome was recorded cancer status for malignant neoplasms of the lip, oral cavity, and pharynx. This outcome was defined using ICD-10 codes C00-C14. Patients were classified as having recorded LOCP cancer if at least one C00-C14 diagnosis code was observed in any available record (7, 16). Because multiple rows were available for some patients, a patient-level binary outcome was generated by taking the maximum value of the row-level C00-C14 cancer indicator across all records for each patient.

### 2.3 Patient-level aggregation

All analyses were performed after converting the dataset to one row per patient. For variables expected to be stable within patients, including sex, age, and entry year, within-patient consistency was checked before aggregation. Within-patient consistency checks showed no discordant coding for sex or entry year among non-missing values. One patient had missing sex information. Age did not vary across repeated records among patients with available age data, but 14 patients had missing age information. These patients were included in descriptive outcome totals where possible but excluded from regression models requiring age category.

### 2.4 Comorbidity variables

Comorbidities were identified using ICD-10 diagnosis codes and aggregated to the patient level. Type 2 diabetes mellitus was defined using E11 codes, obesity using E66 codes, dyslipidemia using E78 codes, gastroesophageal reflux disease (GERD) using K21 codes, and gingival and periodontal disease using K05-related codes. Myocardial infarction was defined using the presence of either I21 or I22 codes. A patient was classified as positive for a comorbidity if at least one corresponding ICD-10 code was present in any of their records.

### 2.5 Laboratory biomarkers

The evaluated laboratory biomarkers included inflammatory, hematological, hepatic, and renal markers. The main biomarkers considered were C-reactive protein (CRP; mg/L), hemoglobin (g/L), red cell distribution width (RDW; %), white blood cell count (WBC; 10⁹/L), glutamate-pyruvate transaminase (GPT; U/L), creatinine (µmol/L), and lactate dehydrogenase (LDH; U/L).

Because multiple records were available for some patients, laboratory variables were first summarized at the patient level using the median of all available values for each biomarker. The median was selected to reduce the influence of repeated measurements and extreme values. Because several biomarkers had skewed distributions and contained implausible or extreme values, plausibility-based cleaning was performed after patient-level aggregation and before transformation. Values outside broad biologically plausible ranges were set to missing. The following ranges were used: hemoglobin 30–250 g/L, RDW 5%–40%, WBC 0.5–200 10⁹/L, GPT 1–2,000 U/L, creatinine 10–1,500 µmol/L, CRP 0–500 mg/L, and LDH 50–5,000 U/L. These thresholds were selected to remove clear technical or data-entry artifacts while retaining clinically extreme values.

Skewed biomarkers were log-transformed before regression modeling. CRP was transformed as ln(CRP+1) to allow inclusion of zero values, while WBC, GPT, creatinine, and LDH were transformed using the natural logarithm after plausibility-based cleaning. Biomarker variables were then standardized using z-scores calculated from the available values of each cleaned and, where applicable, transformed biomarker. Therefore, odds ratios from biomarker models represent the change in odds of C00-C14 cancer status per one-standard-deviation increase in the corresponding biomarker.

Missing biomarker data were not imputed. Biomarker models were fitted using complete-case analysis, restricted to patients with non-missing values for all biomarkers and covariates included in the corresponding model. The core biomarker model included 243,325 patients, including 1,810 LOCP cancer cases. The inflammatory biomarker model included 193,053 patients, including 1,494 LOCP cancer cases.

### 2.6 Statistical analysis

#### 2.6.1 Descriptive analysis

Descriptive analyses were performed at the patient level. Categorical variables were summarized as frequencies and column percentages according to LOCP cancer status. Continuous variables were summarized using medians and interquartile ranges because of skewed biomarker distributions. Pearson's chi-squared tests were used to compare categorical variables between patients with and without LOCP cancer. Wilcoxon rank-sum tests were used to compare continuous variables between groups. All analyses were performed using Stata 19 (17). Statistical significance was assessed using two-sided tests, and *p*-values below 0.05 were considered statistically significant. For table presentation, *p*-values below 0.001 were reported as *p* < 0.001.

#### 2.6.2 Multivariable logistic regression

Multivariable logistic regression was used to estimate adjusted odds ratios and 95% confidence intervals for LOCP cancer status. Robust standard errors were applied in all logistic regression models to reduce sensitivity to model-based variance assumptions.

The primary outcome was patient-level LOCP cancer status.

Four main models were constructed.

This staged strategy was used because biomarker availability differed and each model addressed a distinct analytical objective: clinical associations in the full cohort, associations of the core and inflammatory biomarker blocks in their respective complete-case subsets, and the shape of the CRP dose-response relationship.

The first model was the base clinical model. This model included age category, sex, type 2 diabetes mellitus, obesity, dyslipidemia, myocardial infarction, gastroesophageal reflux disease, gingival/periodontal disease, and entry year. This model was fitted in the full patient-level dataset.

The second model was the core biomarker model. This model included the same clinical covariates as the base model and additionally included hemoglobin, RDW, log-transformed WBC, log-transformed GPT, and log-transformed creatinine. This model was restricted to patients with complete data for all included biomarkers and covariates.

The third model was the inflammatory biomarker model. This model included the same clinical covariates as the base model and additionally included hemoglobin, RDW, log-transformed WBC, log-transformed CRP, and log-transformed LDH. This model was restricted to patients with complete data for the inflammatory biomarker set and covariates.

The fourth model was the CRP quartile model. In this model, CRP was categorized into quartiles based on log-transformed cleaned CRP values among patients in the inflammation-complete subset. The lowest CRP quartile served as the reference category. The model was adjusted for age category, sex, type 2 diabetes mellitus, obesity, dyslipidemia, myocardial infarction, gastroesophageal reflux disease, gingival/periodontal disease, hemoglobin, RDW, log-transformed WBC, log-transformed LDH, and entry year.

Entry-year coefficients were included in all models but were not displayed in the main regression tables to improve readability. The reference categories were female sex, age 18–44 years, absence of each comorbidity, and entry year 2007.

CRP dose-response and trend analysis.

To evaluate whether CRP showed a graded association with LOCP cancer status, CRP quartiles were modeled as categorical predictors using the lowest quartile as the reference category. In addition, a trend analysis was performed by modeling CRP quartile as an ordinal continuous variable. The resulting odds ratio represented the change in odds of LOCP cancer per one-quartile increase in CRP.

#### 2.6.3 Model performance assessment

Model performance was assessed using pseudo-R^2^ (18), Akaike information criterion (19), Bayesian information criterion (20), and the area under the receiver operating characteristic curve (AUC) (21). Model discrimination was evaluated using AUC. Because biomarker models were fitted in complete-case subsets that differed from the full clinical dataset, performance metrics were interpreted with attention to the analytic sample used for each model.

For evaluating the incremental value of biomarker blocks, restricted base models were also fitted in the corresponding biomarker-complete and inflammation-complete subsets. These restricted base models were used to assess whether the addition of biomarkers improved model fit and discrimination within the same analytic sample.

#### 2.6.4 Sensitivity and diagnostic checks

Before finalizing the biomarker models, several diagnostic and sensitivity checks were performed. Within-patient consistency was checked for age, sex, and entry year. Biomarker availability was evaluated at the patient level to determine the sample sizes available for biomarker analyses. Correlations between biomarkers were examined, particularly between GOT and GPT, because of their biological relatedness. Variance inflation factors were assessed using a linear regression diagnostic model as a standard multicollinearity check among predictors (22). GOT and GPT were not included together in the final main biomarker model because sensitivity analyses indicated conditional suppression effects when both were entered simultaneously. GPT was retained in the core biomarker model because it provided a simpler and more interpretable specification.

## 3 Results

The source dataset contained 805,989 records from 333,807 unique patients, with a median of 2 records per patient [IQR: 1–3]. Overall, 1,963 patients (0.59%) had recorded LOCP cancer status. These patients were older, predominantly male, and more frequently had several recorded comorbidities, particularly gingival/periodontal disease and myocardial infarction. Descriptively, LOCP cancer status was characterized by substantially higher CRP, modestly higher WBC and RDW, and lower hemoglobin; complete demographic, comorbidity, and laboratory comparisons are presented in Table 1.

**Table 1:** Patient characteristics by LOCP cancer status.

Variable	No LOCP cancer	LOCP cancer	*p*-value
Total, *n* (%)	331,844 (99.41)	1,963 (0.59)	—
Sex, *n* (%)			<0.001
Male	142,444 (42.93)	1,476 (75.19)	
Female	189,399 (57.07)	487 (24.81)	
Age category, *n* (%)			<0.001
0–17 years	50,930 (15.35)	8 (0.41)	
18–44 years	114,407 (34.48)	126 (6.42)	
45–64 years	90,703 (27.33)	1,207 (61.52)	
≥65 years	75,791 (22.84)	621 (31.65)	
Age, years, median [IQR]	45 [25–63]	60 [53–67]	<0.001
Type 2 diabetes mellitus, *n* (%)	29,390 (8.86)	260 (13.25)	<0.001
Obesity, *n* (%)	21,572 (6.50)	102 (5.20)	0.019
Dyslipidemia, *n* (%)	33,900 (10.22)	282 (14.37)	<0.001
Myocardial infarction, *n* (%)	28,663 (8.64)	370 (18.85)	<0.001
GERD, *n* (%)	24,366 (7.34)	246 (12.53)	<0.001
Gingival/periodontal disease, *n* (%)	1,237 (0.37)	56 (2.85)	<0.001
CRP, mg/L, median [IQR]	6.50 [2.50–22.90]	26.19 [7.00–78.30]	<0.001
Hemoglobin, g/L, median [IQR]	137 [126–148.5]	133 [119–145]	<0.001
RDW, %, median [IQR]	14.40 [13.30–15.50]	14.93 [13.95–15.90]	<0.001
WBC, 10⁹/L, median [IQR]	7.84 [6.41–9.74]	8.23 [6.65–10.33]	<0.001
GPT, U/L, median [IQR]	20 [14.5–29.5]	18.5 [13.5–26.5]	<0.001
Creatinine, µmol/L, median [IQR]	77 [65–91.5]	79.5 [67–95]	<0.001
LDH, U/L, median [IQR]	332 [282.5–401.5]	324.25 [274–393]	0.001

Values are presented as *n* (%) for categorical variables and median [interquartile range] for continuous variables. Percentages are column percentages within LOCP cancer status. LOCP cancer status refers to ICD-10 malignant neoplasms of the lip, oral cavity, and pharynx. Categorical variables were compared using Pearson's chi-squared tests, and continuous variables were compared using Wilcoxon rank-sum tests. GERD, gastroesophageal reflux disease; CRP, C-reactive protein; RDW, red cell distribution width; WBC, white blood cell count; GPT, glutamate-pyruvate transaminase; LDH, lactate dehydrogenase. Minor differences in denominators across variables reflect missing values; age category was available for 333,793 patients and sex for 333,806 patients.

In the full patient-level clinical model displayed in Table 2, age category, sex, GERD, myocardial infarction, and K05-coded gingival/periodontal disease were positively associated with LOCP cancer status, whereas type 2 diabetes mellitus, obesity, and dyslipidemia showed inverse adjusted associations. Compared with patients aged 18–44 years, those aged 45–64 years had markedly higher odds of LOCP cancer (OR=10.67, 95% CI: 8.86–12.86), as did those aged ≥65 years (OR=7.27, 95% CI: 5.97–8.85). Male sex was strongly associated with LOCP cancer status (OR=3.95, 95% CI: 3.56–4.38). Gingival/periodontal disease showed the strongest comorbidity association, with more than eightfold higher adjusted odds of LOCP cancer (OR=8.23, 95% CI: 6.16–10.98). GERD (OR=1.37, 95% CI: 1.19–1.57) and myocardial infarction (OR=1.22, 95% CI: 1.08–1.38) were also positively associated with LOCP cancer status. Type 2 diabetes mellitus (OR=0.84, 95% CI: 0.73–0.97), obesity (OR=0.67, 95% CI: 0.54–0.82), and dyslipidemia (OR=0.80, 95% CI: 0.70–0.92) were inversely associated in the base clinical model. The base clinical regression model included 333,793 patients because 14 patients had missing age information required for age-category classification.

**Table 2:** Multivariable logistic regression models for LOCP cancer status.

Predictor	Model 1: Base clinical model OR [95% CI]	Model 2: Core biomarker model OR [95% CI]	Model 3: Inflammatory biomarker model OR [95% CI]
**N**	333,793	243,325	193,053
**LOCP cancer cases**	1,962	1,810	1,494
**Age category**			
0–17 years	**0.12 [0.06–0.25]**	**0.06 [0.03–0.14]**	**0.14 [0.05–0.39]**
18–44 years	Reference	Reference	Reference
45–64 years	**10.67 [8.86–12.86]**	**8.87 [7.30–10.79]**	**7.03 [5.69–8.69]**
≥65 years	**7.27 [5.97–8.85]**	**4.62 [3.73–5.71]**	**3.36 [2.66–4.24]**
Male sex (ref: female)	**3.95 [3.56–4.38]**	**5.29 [4.70–5.96]**	**3.67 [3.24–4.14]**
Type 2 diabetes mellitus (ref: absent)	**0.84 [0.73–0.97]**	**0.83 [0.72–0.96]**	**0.73 [0.62–0.85]**
Obesity (ref: absent)	**0.67 [0.54–0.82]**	**0.72 [0.58–0.90]**	**0.67 [0.53–0.84]**
Dyslipidemia (ref: absent)	**0.80 [0.70–0.92]**	0.96 [0.84–1.11]	1.01 [0.87–1.17]
Myocardial infarction (ref: absent)	**1.22 [1.08–1.38]**	1.11 [0.99–1.26]	**1.21 [1.06–1.37]**
GERD (ref: absent)	**1.37 [1.19–1.57]**	**1.27 [1.10–1.46]**	**1.34 [1.15–1.55]**
Gingival/periodontal disease (ref: absent)	**8.23 [6.16–10.98]**	**8.82 [6.57–11.84]**	**8.39 [6.08–11.58]**
Hemoglobin, per 1-SD increase	—	**0.72 [0.68–0.75]**	**0.74 [0.71–0.79]**
RDW, per 1-SD increase	—	1.03 [0.98–1.08]	1.01 [0.95–1.06]
WBC, per 1-SD increase in log-transformed value	—	**1.12 [1.08–1.17]**	1.01 [0.96–1.06]
GPT, per 1-SD increase in log-transformed value	—	**0.69 [0.65–0.73]**	—
Creatinine, per 1-SD increase in log-transformed value	—	**0.77 [0.73–0.82]**	—
CRP, per 1-SD increase in log-transformed value	—	—	**1.55 [1.47–1.65]**
LDH, per 1-SD increase in log-transformed value	—	—	**0.80 [0.75–0.85]**

Values are odds ratios with 95% confidence intervals from multivariable logistic regression models with robust standard errors. Model 1 included age category, sex, type 2 diabetes mellitus, obesity, dyslipidemia, myocardial infarction, GERD, gingival/periodontal disease, and entry year. Model 2 included the Model 1 covariates plus hemoglobin, RDW, WBC, GPT, and creatinine. Model 3 included the Model 1 covariates plus hemoglobin, RDW, WBC, CRP, and LDH. Biomarker predictors were cleaned using biologically plausible ranges, log-transformed where appropriate, and standardized; their odds ratios represent the change in odds per one-standard-deviation increase. GERD, gastroesophageal reflux disease; RDW, red cell distribution width; WBC, white blood cell count; GPT, glutamate-pyruvate transaminase; CRP, C-reactive protein; LDH, lactate dehydrogenase. All models were adjusted for entry year, but entry-year coefficients are not displayed.

Bold indicates statistical significance *p*<0.05.

In the core biomarker model, lower hemoglobin, higher WBC, lower GPT, and lower creatinine were independently associated with LOCP cancer status as outlined in Table 2. A one-standard-deviation increase in hemoglobin was associated with lower odds of LOCP cancer (OR=0.72, 95% CI: 0.68–0.75), while a one-standard-deviation increase in log-transformed WBC was associated with higher odds (OR=1.12, 95% CI: 1.08–1.17). RDW was not independently associated with LOCP cancer status in this model.

In the inflammatory biomarker model shown in Table 2, CRP was the strongest biomarker correlate of LOCP cancer. A one-standard-deviation increase in log-transformed CRP was associated with 55% higher adjusted odds of LOCP cancer (OR=1.55, 95% CI: 1.47–1.65). Hemoglobin remained inversely associated with LOCP cancer status (OR=0.74, 95% CI: 0.71–0.79), whereas RDW and WBC were not independently associated after adjustment for CRP and other covariates. LDH showed an inverse association with LOCP cancer status (OR=0.80, 95% CI: 0.75–0.85), which should be interpreted cautiously given its skewed distribution.

Biomarker availability varied across laboratory parameters. The core biomarker model included 243,325 patients with complete data for hemoglobin, RDW, WBC, GPT, creatinine, and covariates, including 1,810 LOCP cancer cases. The inflammatory biomarker model included 193,053 patients with complete data for hemoglobin, RDW, WBC, CRP, LDH, and covariates, including 1,494 LOCP cancer cases.

**Figure 1:**
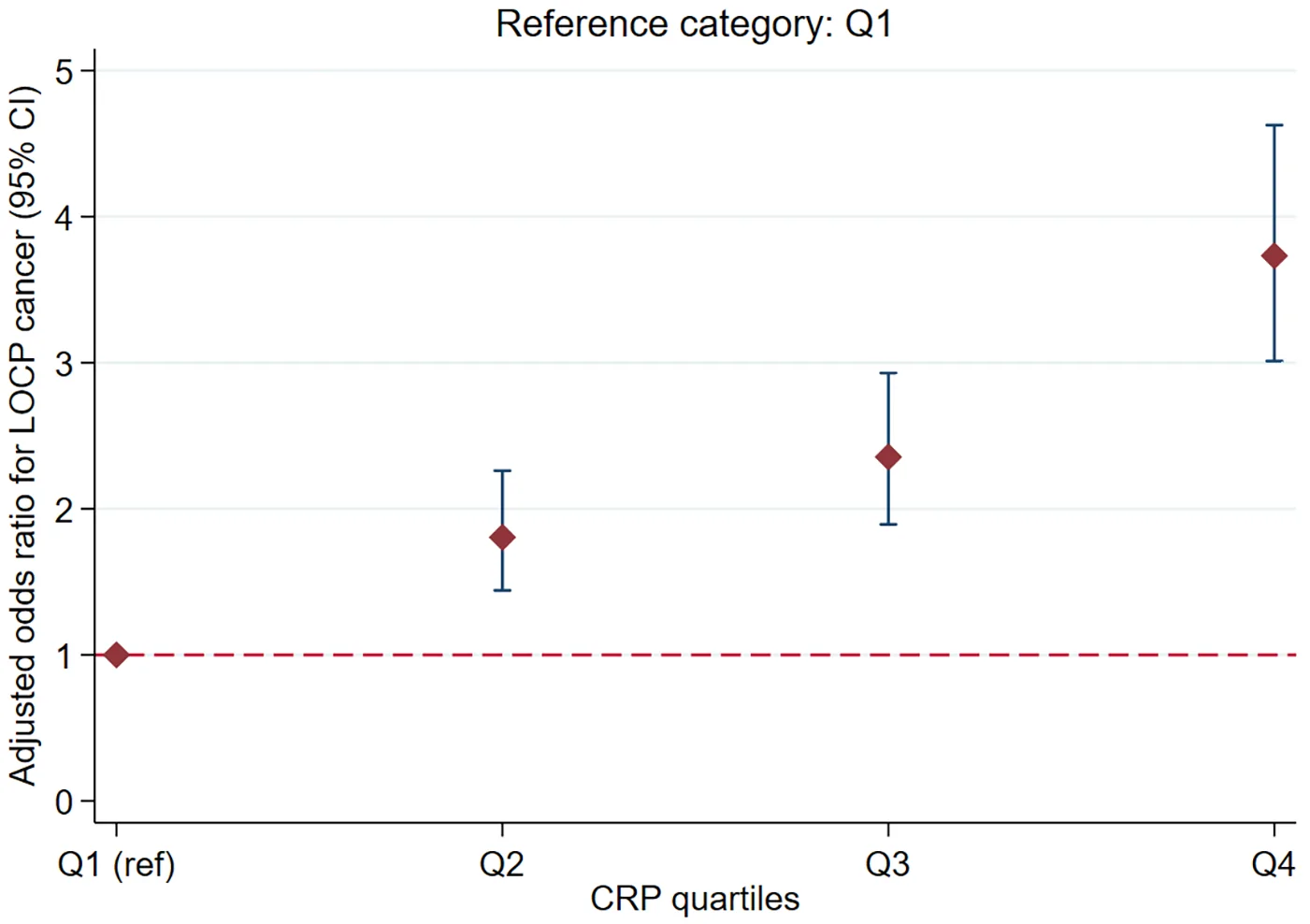
Adjusted association between CRP quartiles and LOCP cancer status. Adjusted odds ratios and 95% confidence intervals were derived from the multivariable logistic regression model adjusted for age category, sex, type 2 diabetes mellitus, obesity, dyslipidemia, myocardial infarction, gastroesophageal reflux disease, gingival/periodontal disease, hemoglobin, red cell distribution width, white blood cell count, lactate dehydrogenase, and entry year. The lowest CRP quartile served as the reference category.

A secondary CRP quartile analysis demonstrated a clear dose-response pattern shown in Table 3 and Figure 1. Compared with patients in the lowest CRP quartile, adjusted odds of LOCP cancer increased progressively across quartiles: Q2 OR=1.81 (95% CI: 1.44–2.26), Q3 OR=2.35 (95% CI: 1.89–2.93), and Q4 OR=3.73 (95% CI: 3.01–4.63). The ordinal trend model confirmed this gradient, with a 51% increase in LOCP cancer odds per quartile increase in CRP (OR=1.51, 95% CI: 1.42–1.60; *p* < 0.001).

**Table 3:** CRP quartile dose-response model for LOCP cancer status.

CRP quartile	CRP range, mg/L	Adjusted OR [95% CI]	*p*-value
Q1, lowest	0.00–2.70	Reference	—
Q2	>2.70–7.30	1.81 [1.44–2.26]	<0.001
Q3	>7.30–27.10	2.35 [1.89–2.93]	<0.001
Q4, highest	>27.10–488.40	3.73 [3.01–4.63]	<0.001
Per-quartile trend	—	1.51 [1.42–1.60]	<0.001

The CRP quartile model was adjusted for age category, sex, type 2 diabetes mellitus, obesity, dyslipidemia, myocardial infarction, GERD, gingival/periodontal disease, hemoglobin, RDW, WBC, LDH, and entry year. CRP quartiles were based on log-transformed cleaned CRP values among patients in the inflammation-complete subset. The trend estimate was obtained by modeling CRP quartile as an ordinal variable. Although quartiles were generated from log-transformed cleaned CRP values, ranges are presented in the original mg/L scale for clinical interpretability. The model was adjusted for entry year, but entry-year coefficients are not displayed.

Restricted base clinical models were fitted in the corresponding complete-case subsets to allow same-sample comparison of model performance. In the biomarker-complete subset, addition of the core biomarker panel increased the AUC from 0.8062 to 0.8287 and reduced the AIC from 19,036.36 to 18,422.53. In the inflammation-complete subset, addition of inflammatory biomarkers increased the AUC from 0.7994 to 0.8243 and reduced the AIC from 15,695.90 to 15,140.25 (Table 4; Supplementary Table 1).

**Table 4:** Model performance metrics for multivariable logistic regression models of LOCP cancer status.

Model	*N*	LOCP cancer cases	Pseudo R^2^	AIC	BIC	AUC
Base clinical model, full sample	333,793	1,962	0.1266	21,072.71	21,351.39	0.8287
Base clinical model, biomarker-complete subset	243,325	1,810	0.1107	19,036.36	19,306.81	0.8062
Core biomarker model	243,325	1,810	0.1400	18,422.53	18,745.00	0.8287
Base clinical model, inflammation-complete subset	193,053	1,494	0.1062	15,695.90	15,960.34	0.7994
Inflammatory biomarker model	193,053	1,494	0.1385	15,140.25	15,455.54	0.8243
CRP quartile model	193,053	1,494	0.1380	15,153.39	15,489.02	0.8242

Pseudo R^2^, Akaike information criterion (AIC), Bayesian information criterion (BIC), and area under the receiver operating characteristic curve (AUC) were used to summarize model fit and discrimination. LOCP cancer status refers to ICD-10 malignant neoplasms of the lip, oral cavity, and pharynx. The base clinical model included age category, sex, type 2 diabetes mellitus, obesity, dyslipidemia, myocardial infarction, gastroesophageal reflux disease, gingival/periodontal disease, and entry year. The core biomarker model additionally included hemoglobin, red cell distribution width, white blood cell count, glutamate-pyruvate transaminase, and creatinine. The inflammatory biomarker model additionally included hemoglobin, red cell distribution width, white blood cell count, C-reactive protein, and lactate dehydrogenase. The CRP quartile model replaced continuous log-transformed CRP with CRP quartiles. Biomarker models were fitted in complete-case subsets; therefore, model performance metrics should be interpreted with attention to differences in analytic sample size.

## 4 Discussion

In this patient-level cross-sectional aggregation of 333,807 unique patients from 805,989 clinical records at University of Debrecen, including 1,963 patients with recorded ICD-10 C00-C14 status, the most coherent biomarker signal was systemic inflammation. Log-transformed CRP showed the clearest independent association with recorded lip, oral cavity, and pharyngeal cancer status, with a strong monotonic quartile gradient, while lower hemoglobin also remained independently associated. Recorded gingival/periodontal disease showed a very large association across models, whereas RDW and WBC differences were largely absorbed after CRP entered the model. Biomarkers improved discrimination only modestly beyond the clinical base model. Taken together, these findings indicate an interconnected pattern of systemic inflammation, hematological compromise, and oral inflammatory burden rather than isolated biomarker effects.

### 4.1 CRP and systemic inflammation

The CRP result is directionally well aligned with the head-and-neck literature. Chen et al.'s meta-analytic signal for HNSCC has been summarized as a pooled overall-survival HR of about 1.84 (6), and Zhang and Gu reported in 208 HNSCC patients that pretreatment CRP >11.3 mg/L independently predicted worse overall survival (HR 1.90, 95% CI 1.32–2.73) and progression-free survival (HR 1.75, 95% CI 1.25–2.45) (23). A newer oral-cancer meta-analysis likewise found worse overall survival (HR 1.80, 95% CI 1.11–2.92) and disease-free survival (HR 1.81, 95% CI 1.28–2.56) among patients with high CRP (24). The convergence across site-specific and broader HNSCC studies supports the present finding as part of a larger, reproducible inflammation signal rather than an isolated database artifact.

That said, the literature is not uniformly positive. Kruse et al., in 278 oral SCC patients, found no significant relation between elevated preoperative CRP and recurrence or metastases (25), and some earlier oral-cavity studies suggested stronger links with pathologic aggressiveness than with downstream events (26, 27). This heterogeneity is not surprising: CRP associations vary by endpoint, subsite, treatment context, timing of blood draw, and the extent to which smoking, nutrition, or comorbidity are controlled. The present study's outcome is even broader: recorded C00-C14 cancer status rather than recurrence or survival. That broader endpoint may capture inflammatory comorbidity structure while diluting site-specific tumor biology.

Mechanistically, the CRP finding is also credible. Acute-phase proteins, including CRP, are downstream products of cytokine-driven inflammation, especially IL-6-signaling (28). Cancer-related inflammation is now understood to participate in tumor initiation, progression, immune modulation, tissue remodeling, and cachexia (29). In HNSCC specifically, IL-6-related pathways have repeatedly been implicated in tumor progression and adverse outcomes (30). The present CRP gradient therefore sits well within contemporary models of tumor-host inflammatory interaction.

### 4.2 Hemoglobin, anemia, RDW, and WBC

The inverse hemoglobin association is concordant with a substantial HNC literature linking pretreatment anemia to poorer outcome. Fortin et al. reported that anemia was the strongest predictor of poorer local control and survival during concurrent radiochemotherapy, with hazard ratios of 0.37 for local control and 0.47 for survival in their modeling framework (31). More recently, Ma et al. identified an HNC chemoradiation threshold of 11.4 g/dL, below which survival outcomes worsened (32), and Kürten et al. showed that even mild pretreatment anemia was associated with inferior overall survival, with 64% versus 85% survival in mildly anemic versus non-anemic patients (33). The present cross-sectional result does not establish prognosis, but it fits the same biological and clinical axis: lower hemoglobin may mark a less favorable host-tumor or systemic illness state.

Biologically, that association can arise through multiple, non-mutually exclusive pathways: anemia of chronic inflammation through IL-6-hepcidin signaling and iron restriction, nutritional deficiency, occult bleeding, marrow suppression, renal dysfunction, or generalized catabolic illness (34). This multiplicity is important because it argues against a simplistic “tumor causes anemia” reading. In head and neck oncology, hemoglobin often functions as an integrated marker of inflammatory burden, nutritional compromise, and tissue hypoxia, the latter being particularly relevant to radiosensitivity in prior prognostic studies (35).

By contrast, the attenuation of RDW and WBC after CRP adjustment is also interpretable and literature-consistent. Tham et al.'s meta-analysis reported pooled adverse associations for elevated RDW in upper aerodigestive tract cancers, including overall-survival HR 1.44 and recurrence-free-survival HR 1.43 (36), and recent OSCC work has found RDW to remain independently associated with lower survival, for example HR 1.541 in the 2023 Trevisani et al. cohort (37). Yet RDW is biologically downstream of inflammation, impaired erythropoiesis, iron dysregulation, oxidative stress, and malnutrition; once CRP and hemoglobin are modeled simultaneously, little unique information may remain (38).

Evidence concerning total WBC is inconsistent across settings. In 278 patients with oral cancer, Kruse et al. found no significant association between preoperative WBC count and recurrence or metastases (39). The present pattern, showing descriptively higher WBC, loss of independent signal after CRP fits a parsimonious interpretation: total WBC is too nonspecific in this setting, and much of its information overlaps with the acute-phase response. Differential counts or composite inflammatory indices may provide more specific information than total WBC in subsequent analyses, particularly if neutrophil, lymphocyte, platelet, and systemic immune-inflammation indices are available.

### 4.3 Periodontal disease and oral inflammatory burden

The magnitude of the gingival/periodontal disease association in the present study was larger than most published estimates. Published pooled estimates are materially smaller: Zeng et al.'s meta-analysis of head and neck cancer reported OR 2.63 (95% CI 1.68–4.14) (40), and Ma et al.'s 2024 oral-cancer meta-analysis reported OR 2.94 (95% CI 2.13–4.07) (41). Individual studies, including Tezal et al.'s tongue-cancer work and the 2019 Shin et al. case-control study, also support a positive association, but not usually at the magnitude seen here (42, 43). Thus, the current OR around 8.4 should be presented as stronger than expected from the literature, not simply “consistent” with it. The unusually large estimate may additionally reflect selective K05 coding of clinically recognized periodontal disease, greater dental and healthcare contact among cancer patients, reverse causation, and residual confounding rather than a direct causal effect of this magnitude.

A biologically plausible explanation certainly exists. Chronic periodontitis can sustain local and systemic inflammation, alter epithelial barrier integrity, shift the oral microbiome, and expose tissues to carcinogenesis-relevant organisms such as Porphyromonas gingivalis (44, 45). Experimental and mechanistic work suggests that P. gingivalis can promote invasion, matrix remodeling, inflammatory signaling, and a tumor-supportive microenvironment in OSCC (46). However, even recent reviews conclude that the causal chain remains incompletely resolved (10). For that reason, the present finding is best interpreted as a powerful epidemiologic correlate of recorded cancer status, not as proof of an eight-fold causal effect of periodontal disease on LOCP cancer.

### 4.4 Metabolic comorbidity paradox and inverse biochemical associations

The inverse adjusted associations for type 2 diabetes and obesity, and the attenuation of dyslipidemia after biomarker adjustment, warrant a deliberately cautious discussion. Meta-analytic work suggests that diabetes is associated with increased oral-cancer risk, with pooled OR around 1.41, and population-based studies have linked diabetes with increased head-and-neck-cancer incidence across several subsites (47). Likewise, the supposed “protective” relation of higher BMI in head and neck cancer has long been recognized as vulnerable to smoking confounding and reverse causation: in pooled cohort data, higher BMI was associated with increased HNC risk in never-smokers (HR 1.15 per 5 kg/m^2^) but decreased risk in current smokers (HR 0.76 per 5 kg/m^2^) (48). Thus, the inverse metabolic pattern in the present manuscript is more plausibly non-causal than biologically protective.

The inverse adjusted associations for GPT, creatinine, and LDH should be read in the same spirit. Low ALT/GPT has increasingly been discussed as a frailty/sarcopenia marker rather than simply a benign laboratory finding (49), and low serum creatinine can likewise track reduced muscle mass and cachexia (50). LDH is more complex: several HNSCC and OSCC studies associate higher LDH with worse outcome, including very large hazard ratios in OSCC, yet at least one resected laryngeal SCC cohort found higher preoperative LDH associated with better survival (51). In a broad, cross-sectional C00-C14 database with no stage, subsite, or treatment timing, these inverse coefficients are therefore better discussed as case-mix-dependent signals than as mechanistic “protection.”

### 4.5 Clinical implications and model performance

From a clinical perspective, the most useful reading of these results is pragmatic. CRP and hemoglobin are inexpensive, routine, scalable measures that appear to capture meaningful information about the host context in which LOCP cancer is recorded. That makes them plausible components of contextual risk-enrichment or supportive triage models in clinical databases. It does not make them suitable as stand-alone screening markers for oral or pharyngeal cancer. CRP is nonspecific, hemoglobin is biologically pleiotropic, and the present design cannot determine whether abnormal values antedate cancer, accompany it, or result from its management.

The AUC results should therefore be interpreted conservatively. An increase of roughly 0.02–0.03 suggests added discriminatory information, but discrimination alone is not enough to judge clinical usefulness. Prediction-model guidance emphasizes that calibration, overfitting assessment, external validation, and decision-analytic performance are all needed; AUC can be informative, but it is insufficient on its own to establish whether a model improves decisions in practice (52). For that reason, the current study will not draw strong translational claims and instead state that biomarkers provide incremental but currently preliminary value for discrimination in this database.

### 4.6 Strengths and limitations

This study has several strengths, including its large real-world clinical sample, patient-level aggregation of repeated records, and use of routinely available laboratory markers with direct clinical relevance. The staged modeling strategy enabled biomarker associations to be evaluated in defined complete-case subsets, while the consistency of the CRP signal across continuous and quartile-based analyses supports the internal robustness of the inflammatory finding.

Several limitations must be acknowledged. First, the cross-sectional patient-level design prevents assessment of temporality or causality; therefore, the findings should be interpreted as correlates of recorded C00-C14 cancer status rather than incident cancer predictors or etiological risk factors. Laboratory abnormalities may reflect cancer-related inflammation, diagnostic workup, comorbidity burden, infection, treatment effects, malnutrition, or healthcare-contact intensity. Second, C00-C14 is a heterogeneous administrative outcome that includes lip, oral cavity, salivary gland, pharyngeal, tonsillar, and ill-defined sites; subsite, histology, stage, HPV status, treatment status, diagnosis date, longitudinal follow-up, and mortality data were unavailable, precluding survival and other time-to-event analyses. Third, residual confounding is likely because the database lacked smoking, alcohol use, socioeconomic status, oral hygiene, dental-care utilization, nutritional status, medication use, and occupational or environmental exposures. This is particularly relevant for the periodontal association and for the inverse adjusted associations observed for type 2 diabetes mellitus, obesity, and dyslipidemia, which should not be interpreted as protective effects. Finally, biomarker testing was performed during routine care rather than by protocol, so missingness and measurement timing were likely non-random. Complete-case biomarker models may therefore reflect clinically selected subgroups. External validation in longitudinal, site-specific datasets with richer behavioral, pathological, treatment, and biomarker-timing information is needed before these findings can be generalized or used for prediction.

## 5 Conclusion

In this large retrospective clinical database, recorded LOCP cancer status was associated with a distinct inflammatory and hematological profile, most consistently reflected by higher CRP and lower hemoglobin. These markers should be interpreted as clinical correlates rather than predictive or etiological indicators until validated in longitudinal, site-specific datasets with richer behavioral and tumor-level information.

## Data Availability

The datasets presented in this article are not readily available because the data that support the findings of this study are available from Clinical Centre of the University of Debrecen, Hungary but restrictions apply to the availability of these data, which were used under license for the current study, and so are not publicly available. Data are, however, available from the authors upon reasonable request and with permission of the Clinical Centre of the University of Debrecen, Hungary. Requests to access the datasets should be directed to nagy.attila@etk.unideb.hu.

## Supplementary material

Supplementary Table 1 Restricted base clinical models fitted in biomarker-complete analytic subsets.
