# Supplementary material for: Clinical and laboratory biomarker correlates of lip, oral cavity, and pharyngeal cancer status in a large retrospective clinical database
Source: Front Oral Health. 2026 Aug 11;7:1888417. doi: 10.3389/froh.2026.1888417 (PMC13503193; doi:10.3389/froh.2026.1888417)
Supplement: Supplementary file 1 [file Table1.docx]

Supplementary Material

Supplementary Table 1. Restricted base clinical models fitted in biomarker-complete analytic subsets

| **Predictor** | **Base model in biomarker-complete subset OR [95% CI]** | **Base model in inflammation-complete subset OR [95% CI]** |
| --- | --- | --- |
| **N** | 243,325 | 193,053 |
| **LOCP cancer cases** | 1,810 | 1,494 |
| **Age category** |  |  |
| 0–17 years | 0.15 [0.06–0.33] | 0.12 [0.05–0.34] |
| 18–44 years | Reference | Reference |
| 45–64 years | 10.06 [8.30–12.19] | 8.86 [7.19–10.91] |
| ≥65 years | 6.62 [5.41–8.11] | 5.50 [4.41–6.85] |
| **Male sex (ref: female)** | 3.70 [3.32–4.13] | 3.71 [3.29–4.18] |
| **Type 2 diabetes mellitus (ref: absent)** | 0.83 [0.72–0.96] | 0.85 [0.73–0.99] |
| **Obesity (ref: absent)** | 0.64 [0.51–0.79] | 0.64 [0.51–0.80] |
| **Dyslipidemia (ref: absent)** | 0.80 [0.70–0.92] | 0.78 [0.67–0.90] |
| **Myocardial infarction (ref: absent)** | 1.15 [1.02–1.31] | 1.16 [1.02–1.32] |
| **GERD (ref: absent)** | 1.30 [1.13–1.50] | 1.31 [1.13–1.53] |
| **Gingival/Periodontal disease (ref: absent)** | 7.94 [5.90–10.68] | 7.45 [5.40–10.29] |
| **Hemoglobin, per 1-SD increase** | Not included | Not included |
| **RDW, per 1-SD increase** | Not included | Not included |
| **WBC, per 1-SD increase in log-transformed value** | Not included | Not included |
| **GPT, per 1-SD increase in log-transformed value** | Not included | Not included |
| **Creatinine, per 1-SD increase in log-transformed value** | Not included | Not included |
| **CRP, per 1-SD increase in log-transformed value** | Not included | Not included |
| **LDH, per 1-SD increase in log-transformed value** | Not included | Not included |

**Footnote**: Values are adjusted odds ratios with 95% confidence intervals from multivariable logistic regression models with robust standard errors. LOCP cancer status refers to ICD-10 malignant neoplasms of the lip, oral cavity, and pharynx. The biomarker-complete subset refers to patients with complete data for the covariates and biomarkers included in the core biomarker model. The inflammation-complete subset refers to patients with complete data for the covariates and biomarkers included in the inflammatory biomarker model. Both restricted base clinical models included age category, sex, type 2 diabetes mellitus, obesity, dyslipidemia, myocardial infarction, gastroesophageal reflux disease, gingival/periodontal disease, and entry year. Entry-year coefficients were included in the models but omitted from the table for readability. Biomarker rows are shown as “Not included” to preserve the same predictor structure as the main regression table. GERD, gastroesophageal reflux disease; RDW, red cell distribution width; WBC, white blood cell count; GPT, glutamate-pyruvate transaminase; CRP, C-reactive protein; LDH, lactate dehydrogenase; SD, standard deviation.
